# Sturge-Weber Syndrome: A Rare Case Report

**DOI:** 10.7759/cureus.28786

**Published:** 2022-09-05

**Authors:** Darshankumar M Raval, Vaishnavi M Rathod, Anjali B Patel, Bhavya Sharma, Princy D Lukhi

**Affiliations:** 1 Department of General Medicine, Sir Sayaji General (SSG) Hospital, Medical College Baroda, Vadodara, IND

**Keywords:** neurocutaneous syndrome, tram-track sign, nevus flammeus, port-wine stain, sturge weber syndrome

## Abstract

Sturge-Weber syndrome (SWS) is a rare sporadic neurocutaneous syndrome characterized by angiomas involving the face, eyes, and brain (leptomeninges). Classical port-wine stains are seen in the ophthalmic and maxillary division of the trigeminal nerve. The most common presenting feature is seizures, the onset of which ranges from birth to late adulthood. Diagnosis is mainly done by brain radio imaging (CT scan and MRI with gadolinium contrast) where characteristic features of calcification and leptomeningeal enhancement are seen.

We report a newly diagnosed case of SWS in a 31-year-old female patient who presented to our hospital with a complaint of generalized tonic-clonic (GTCS) type of convulsion two days prior to the admission with purple discoloration of the skin on the right side of the face, trunk, and right upper limb since birth. During the evaluation of past medical history, the patient was found to have a known case of epilepsy since the age of three months and on was on irregular treatment. To find out the cause of the seizure and skin lesions, further investigations were done which were suggestive of SWS in MRI and CT scanning of the brain. The patient was counseled about the syndrome and discharged on anti-convulsion treatment with advice for dye laser photocoagulation for port-wine stain.

SWS is a rare sporadic genetic disease and diagnosis is primarily done by evaluating history, the presence of port-wine stain, and characteristic features on brain radio imaging. As no definitive treatment is available yet, patients are being treated by medical and surgical interventions for symptoms as well as for associated complications.

## Introduction

Sturge-Weber syndrome (SWS), also known as encephalotrigeminal angiomatosis, is a type of neurocutaneous syndrome with peculiar features such as angiomas on the face, choroid, and leptomeninges. The capillary vascular malformation involving the face is also known as a port-wine stain or nevus flammeus; it is more commonly observed in the area supplied by the trigeminal nerve, rarely extending to the trunk and/or arms. After neurofibromatosis and tuberous sclerosis, this is the third most common neurocutaneous syndrome [1]. The most common presenting neurologic manifestation of SWS is seizure, such as atonic, tonic, or myoclonic types, with the age of onset ranging from birth to 23 years [2]. Although the incidence of this syndrome is not reported accurately, it is estimated to be 1 in 20,000-50,000 live births [1]. There is no gender or race predilection for SWS [1].

Here, we present a newly diagnosed case of SWS in a 31 year-old-female who presented with convulsion with classical port-wine stain involving the face as well as the trunk and upper limb, making this a rare presentation.

## Case presentation

**Figure 1:**
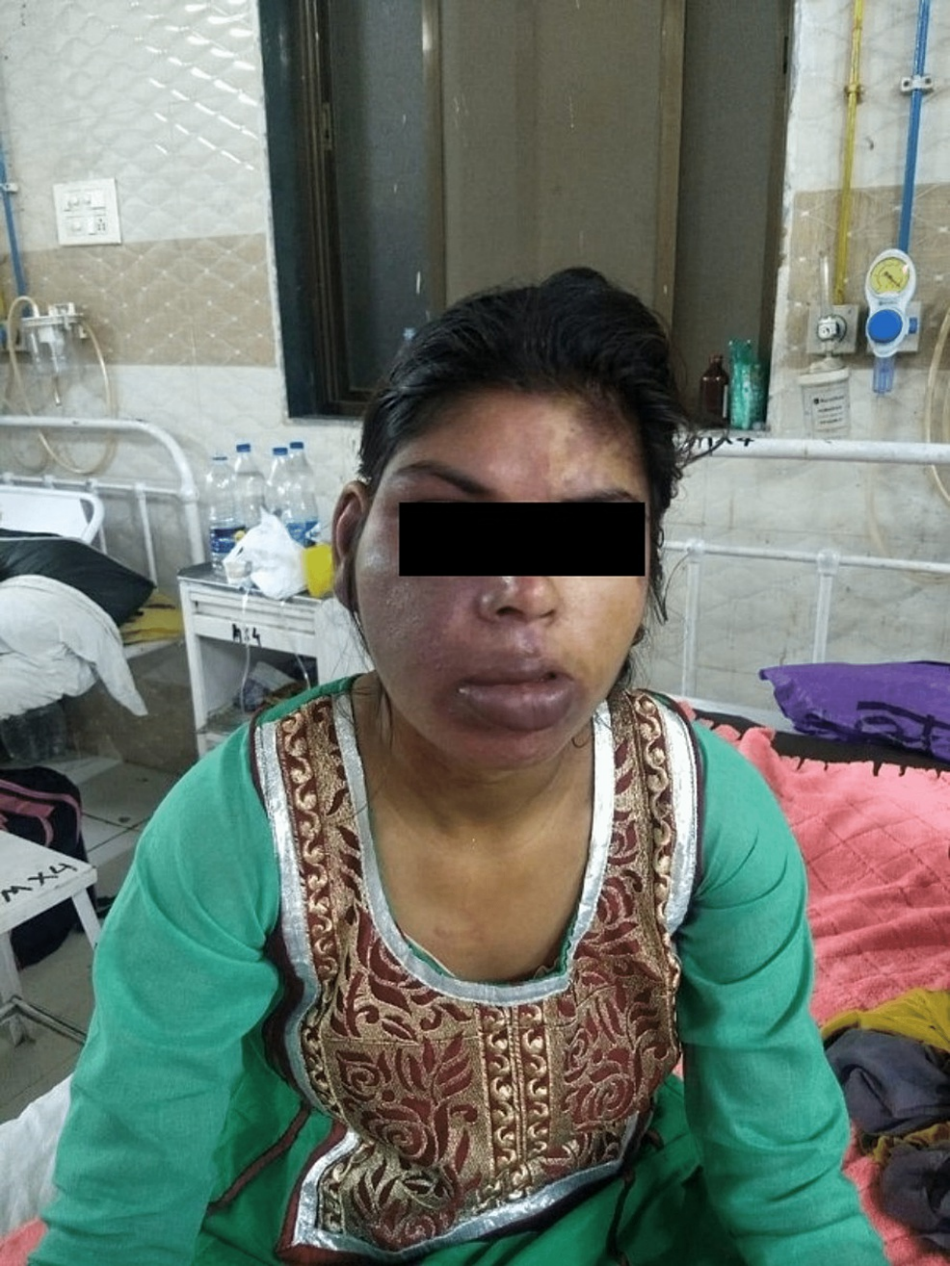
Port-wine stain involving the right side of the face with soft tissue hypertrophy

**Figure 2:**
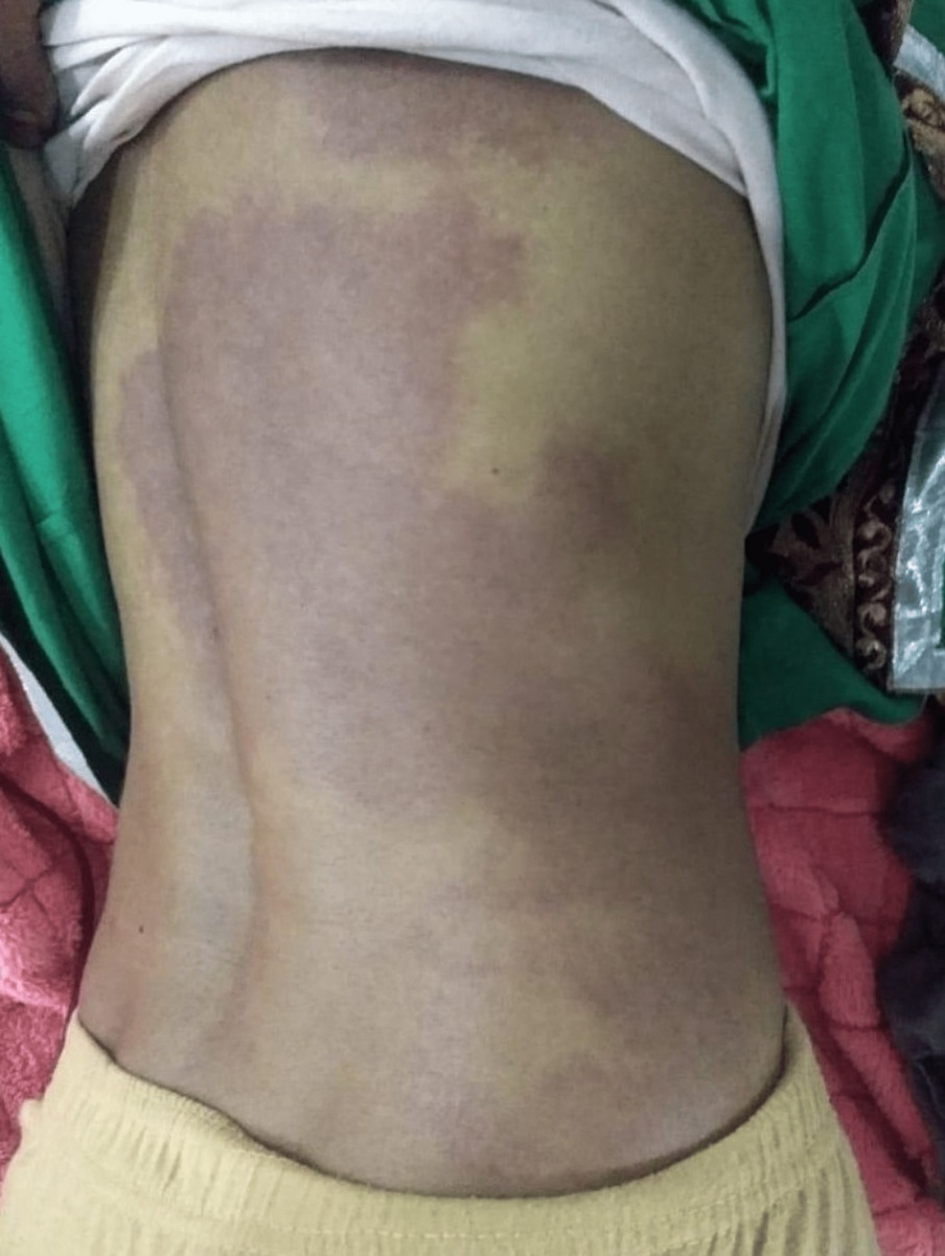
Port-wine stain involving the right side of the trunk

**Figure 3:**
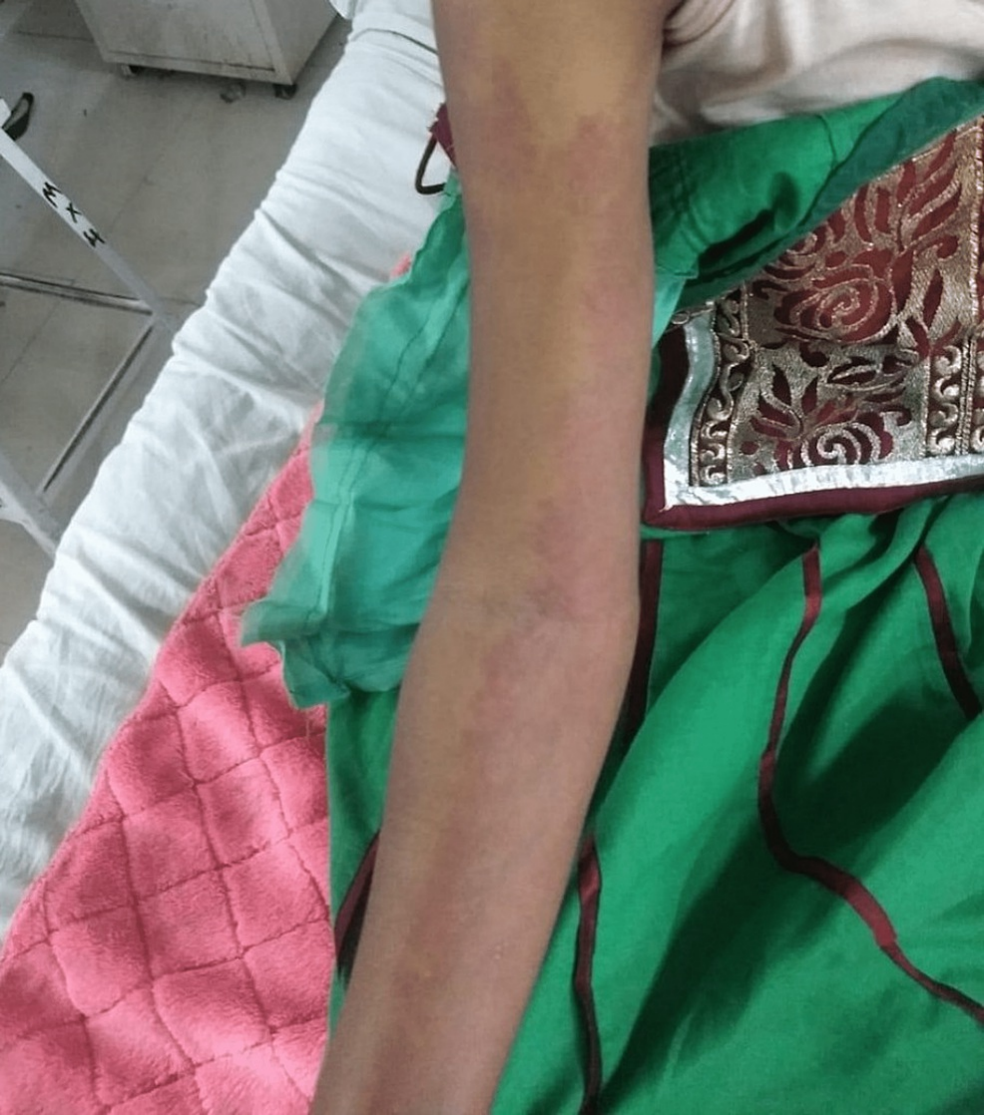
Port-wine stain involving the medial aspect of the right arm and forearm

A 31-year-old unmarried female patient presented to our hospital with complaints of a single episode of generalized tonic-clonic (GTCS) type of convulsion two days prior to admission; it was associated with purple discoloration of the skin of the right side of the face, arm, and forearm (medial aspect), thorax and abdomen since birth, which blanches on pressure. There was no history of fever, headache, vomiting, head trauma, stroke-like events (focal neurological deficit), dizziness, or diminished vision. A review of the patient's medical history revealed that she had her first episode of epilepsy at the age of three months, and since then, she has had multiple episodes of epilepsy while on irregular treatment. There was no significant family, menstrual, obstetric, or personal history. On general examination, the patient was vitally stable with the presence of purple lesions over the skin on the right side of the face in the frontal region, nose, eyelid, cheek, and lips along with hypertrophy of soft tissue. These lesions extended to the skin of the right upper and lower trunk, medial aspect of the right arm and forearm involving the whole wrist, suggestive of port-wine stain (nevus flammeus) (Figures 1-3).

The central nervous system (CNS) examination revealed no abnormality with no focal neurological deficit or cranial nerve involvement; other examinations were normal.

**Figure 4:**
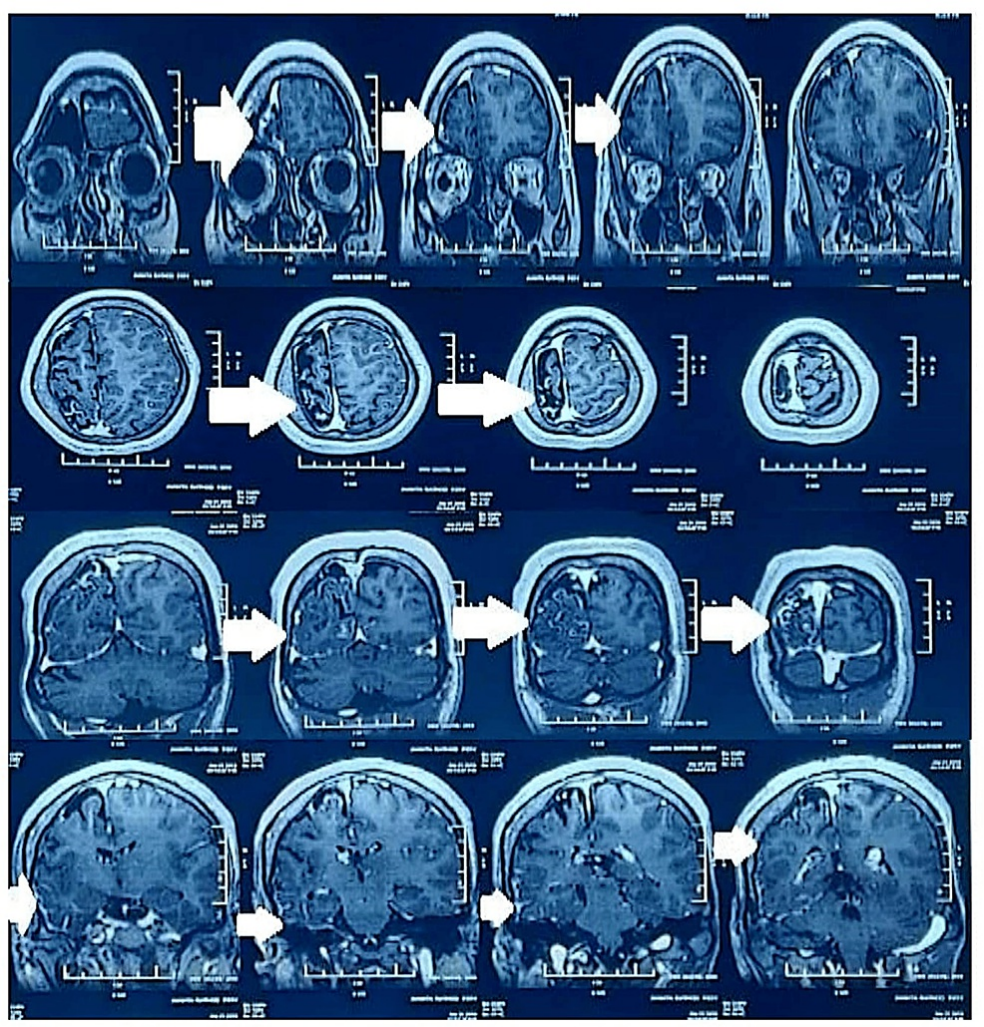
MRI brain (plain + contrast) showing abnormal smooth serpiginous leptomeningeal enhancement in the right parieto-occipito-temporal lobes

Routine and radiological investigations were done to find out the cause of the convulsion and skin lesions (Tables 1-2) (Figure 4).

**Table 1:** Routine blood investigations with C-fundus examination N/L/E/M - Neutrophil/Lymphocyte/Eosinophil/Monocyte

Investigations	Values
Hemoglobin (gm %)	12.5
Total Count (per cumm)	9,300
Differential Count (N/L/E/M %)	54/44/01/01
Platelet Count (per cumm)	3,73,000
Random Blood Sugar (mg/dl)	85
Urea (mg/dl)	25
Creatinine (mg/dl)	0.87
Total Bilirubin (mg/dl)	1.0
Direct Bilirubin (mg/dl)	0.4
Indirect Bilirubin (mg/dl)	0.6
Sodium (mmol/L)	140
Potassium (mmol/L)	4.7
Magnesium (mg/dL)	1.8
Ionized Calcium (mmol/L)	1.31
Urine Routine Micro	Normal
C – Fundus	No Papilledema

**Table 2:** Radiological and other investigations

Investigations	Reports
Ultrasonography of Abdomen & Pelvis	Normal
Skull X-ray	Normal
CT Head (Plain)	Calcification, Tram track appearance, cortical atrophy
MRI Brain (Plain + Contrast)	- Hemi-atrophy of right cerebral hemisphere is seen with multifocal gliotic areas and curvilinear thick blooming image p/o calcification, predominantly in the right parieto-occipito-temporal lobes- Abnormal smooth leptomeningeal enhancement is seen along right parieto-occipito-temporal lobes (as seen in Figure 2) s/o Dyke-Davidoff-Masson syndrome or Sturge-Weber syndrome.
Electroencephalogram (EEG)	Reduced background activity

After considering the history, clinical findings, and investigations, the patient was diagnosed with SWS.

The patient was informed about the syndrome, its prognosis, and complications along with the importance of continuation of anti-convulsant medications. She was discharged with anti-convulsant medications and was advised to undergo dye laser photocoagulation for the port-wine stain.

## Discussion

SWS is part of a group of disorders together known as phakomatoses (“mother-spot” diseases). It comprises congenital hamartomatous malformations, affecting the eye, skin, and CNS inconsistently, presenting as a combination of venous angiomas of the leptomeninges, face, jaws, and oral soft tissues. Angiomas of leptomeninges are commonly seen in the unilateral parietal and occipital region, altering vascular dynamics and resulting in the precipitation of calcium deposits in the cerebral cortex underlying the angioma, which is the causative factor for seizures, mental retardation, hemiplegia, or hemiparesis, depending on the extent of the lesion. The cutaneous angiomas namely port-wine stains or nevus flammeus, usually occur unilaterally along dermatomes supplied by the trigeminal nerve, particularly the ophthalmic and maxillary divisions, where involvement of ophthalmic division is pathognomic. However, port-wine stains can present bilaterally or be totally absent, or may extend to the neck, limbs, and other parts of the body [3]. Glaucoma, choroidal hemangioma, buphthalmos, and hemianopia are known ocular manifestations of the syndrome [3].

Being a sporadic developmental disorder, SWS is a result of somatic mosaic mutations in the GNAQ gene located on the long arm of chromosome 9 [1].

SWS is classified as complete SWS when it involves both CNS and facial angiomas, and incomplete SWS when it involves only one area without the other. According to another scale, namely the Roach scale, SWS is classified into three types [4]: (1) Type I - Both facial and leptomeningeal angiomas; may have glaucoma, (2) Type II - Facial angiomas alone; may have glaucoma, (3) Type III - Isolated leptomeningeal angiomas; usually no glaucoma.

Rendu-Osler-Weber syndrome, Von Hippel-Lindau disease, Maffucci's syndrome, angio-osteodystrophy syndrome, and Klippel Trenaumy-Weber syndrome are the important differential diagnosis for SWS [5]. Rendu-Osler-Weber syndrome is characterized by the presence of multiple telangiectasias in mucocutaneous sites such as the face, lips, tongue, palms, and fingers, as well as the gastrointestinal tract (GI), lungs, brain, lungs, and liver, which can bleed easily. The patient usually presents with nose bleed, GI bleed, iron deficiency anemia, or intracranial bleed in severe cases [6]. Patients with Von Hippel-Lindau disease have hemangioblastomas of the retina, spinal cord, and brain; clear cell renal cell carcinoma and renal cysts; pancreatic cysts; endolymphatic sac tumor; pheochromocytoma, and neuro-endocrine tumors [7]. In Maffucci's syndrome, the patient presents with fractures/deformities due to enchondromatosis. The cavernous hemangiomas of the subcutis, dermis, or internal organs are also present [8]. Congenital vascular bone syndrome or angio-osteodystrophy syndrome is characterized by increased or decreased bone growth due to arterio-venous malformations in long bones [9]. Klippel Trenaumy-Weber syndrome is a triad of bony and soft tissue hypertrophy, mostly atypical lateral varicosity, and capillary malformation (port-wine stain); however, no CNS affection is seen [10]. 

Diagnosis is made by brain radio imaging such as CT scanning and MRI with gadolinium contrast, where classical gyriform calcification (tram-track sign) and serpiginous leptomeningeal enhancement are seen.

There is no specific treatment for SWS. Medical care in SWS includes anticonvulsants for control of seizures, symptomatic and prophylactic treatment for headaches, treatment to reduce intra-ocular pressure in case of glaucoma, and dye laser photocoagulation for port-wine stain. Surgery is preferred in patients with refractory seizures, glaucoma, or specific conditions related to various SWS-associated disorders, such as scoliosis. Surgical interventions for SWS include focal cortical resection, hemispherectomy, corpus callosotomy, vagal nerve stimulation, and operative intervention for diffuse choroidal hemangiomas with retinal detachment and glaucoma. Our patient was also counselled about the importance of regularly taking medications and was discharged with oral anti-convulsion medication. The patient was also advised to undergo dye laser photocoagulation for port wine stain. No surgical management for the seizures was required in our patient as the seizures were occurring due to irregularity in the treatment. 

## Conclusions

SWS is a rare sporadic genetic syndrome for which definitive treatment is not yet available, therefore, at present, it is treated by symptomatic medical and surgical management. We have presented a case of newly diagnosed SWS with port-wine stains extending to the trunk and upper limbs, making this a rare presentation.
